# Isolated MASON type-III radial head fractures: radial head arthroplasty or open reduction and internal fixation – clinical and radiological outcomes with five to fourteen years of follow up

**DOI:** 10.1007/s00264-025-06445-z

**Published:** 2025-02-17

**Authors:** Lyliane Ly, Thibault Druel, Aram Gazarian, Arnaud Walch

**Affiliations:** 1 Hôpital Édouard-Herriot, Lyon, France; 2 Clinique du Parc, Lyon, France

**Keywords:** Radial head fractures, Mason type-III, Arthroplasty, ORIF

## Abstract

**Purpose:**

The aim of this study was to assess functional and radiological outcomes of radial head arthroplasty (RHA) compared to open reduction and internal fixation (ORIF) in isolated Mason type-III fractures with a minimum of five years follow-up.

**Methods:**

This was a retrospective single-center study of closed isolated Mason type-III radial head fractures operated between January 2008 and December 2017. Nineteen patients were included in group RHA and 35 patients in group ORIF. The mean age was 51 years old in group RHA and 41 years old in group ORIF (*p* = 0.02). Functional and radiological outcomes were evaluated.

**Results:**

Mean follow up was eight years (range, 5–14). Clinical results and functional scores showed no significant differences, except a better pronation in group RHA (*p* = 0.04). Two secondary radial head resection or implant removal were performed in each group (*p* = 0.56) with poor functional outcomes in group ORIF. There was less heterotopic ossification in group RHA (15.8% vs. 42.8%; *p* = 0.03). Capitulum wear was found in 63% in group RHA against 25.7% in group ORIF (*p* < 0.05).

**Conclusion:**

Functional results of RHA and ORIF were comparable for isolated Mason type-III fractures at a mean follow-up of eight years. We recommend to perform RHA for isolated Mason type-III fracture if articular reduction or stability of the fixation is not satisfying.

**Level of evidence:**

III.

## Introduction

Radial head fractures (RHF) are the most common fractures of the elbow (33%) [1]. They are usually classified with the Mason classification, divided into three types; type-III being comminuted fractures involving the whole radial head (RH) [2].

Management of type-III fractures remains controversial and challenging. Indeed, complex RHF were resected for a long time, but better comprehension of the stabilizing role of the RH (forearm rotation, stability of elbow, forearm and distal radio-ulnar joint) has changed its management. Joint restoration is key for good functional outcomes [3]. Two main surgical options exist: open reduction and internal fixation (ORIF) and radial head arthroplasty (RHA).

Some studies have assessed outcomes of RHA compared to ORIF for complex RHF and recommend RHA especially when associated with other elbow injuries [4], reported in up to 75% for type-III [5]; but may induce bias in the outcomes of both treatments. Therefore, very few studies have presented clinical and functional outcomes of either RHA or ORIF in isolated Mason type-III fractures [6–8].

The aim of this study was to assess clinical, functional, and radiological results of RHA compared to ORIF in isolated Mason type-III fractures with a minimum of five years follow-up.

## Materials and methods

### Patients

This was a retrospective single-centre study of RHF surgically treated between January 2008 and December 2017. The operative database identified 242 patients who sustained a RHF during the study period. The inclusion criteria were patients of 18 years or older presenting a closed comminuted RHF confirmed by a preoperative CT-scan, surgically treated within 15 days of the traumatism, with a minimum of five years follow-up.

**Fig. 1:**
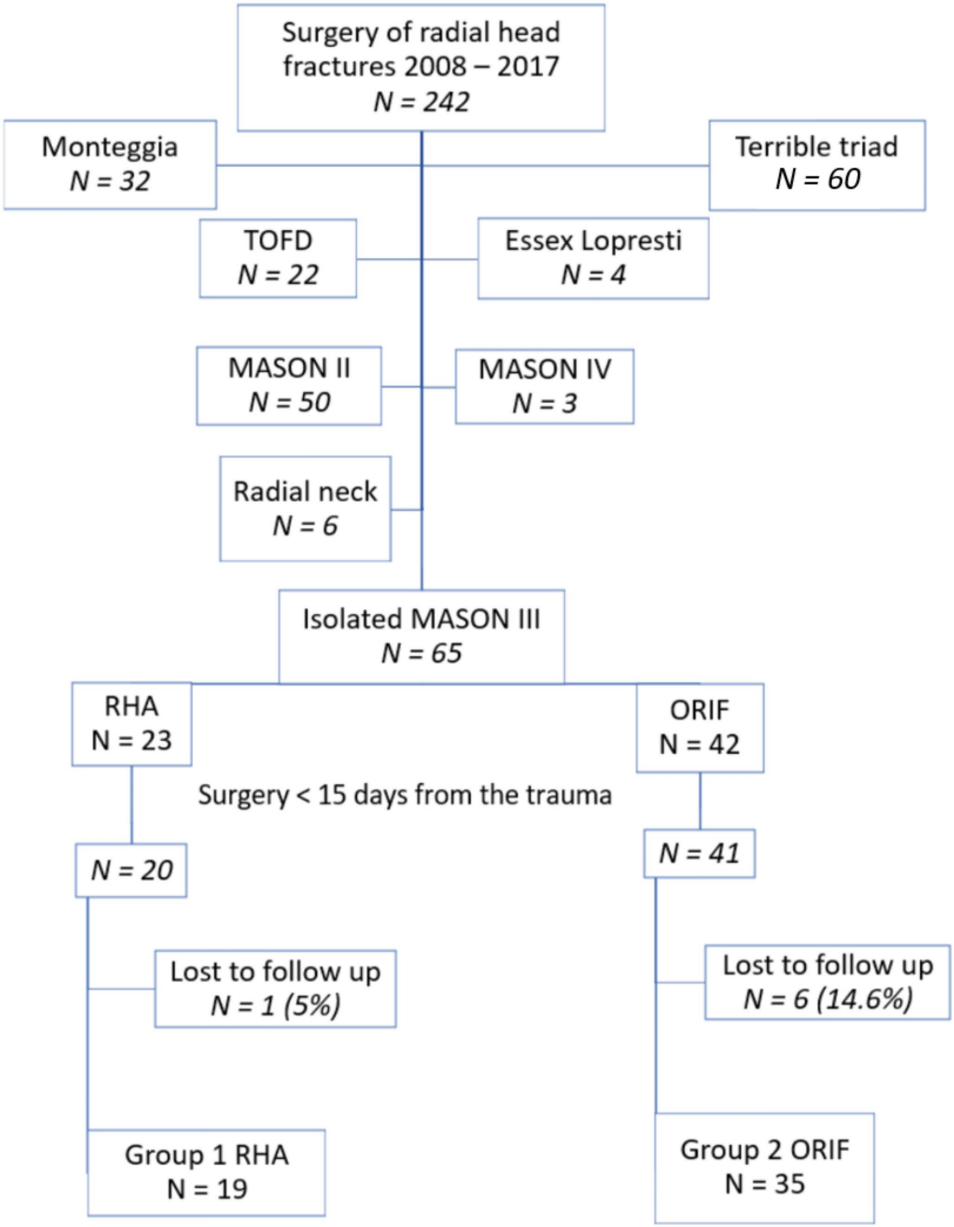
Workflow diagram with details of excluded associated injuries. TOFD: trans olecranon fracture dislocation, RHA: radial head arthroplasty, ORIF: open reduction and internal fixation

All the non-isolated Mason type-III RHF were excluded. Sixty-five type-III fractures remained. Four were treated more than 15 days after the injury, and seven patients were lost to follow-up. A total of 19 patients in group RHA, and 35 in group ORIF were included in the final analysis (Fig. 1).

### Surgical technique

The skin incision was centered on the lateral epicondyle. The RH was exposed through Kocher interval between anconeus and extensor carpi ulnaris muscles, allowing assessment of the lateral collateral ligament (LCL) [9]. There were five (26%) torn LCL in group RHA and three (8.5%) in group ORIF (*p* > 0.05), that were repaired by trans-osseous suture.

The management was done according to the surgeon’s preference. RHA was performed when the fracture was deemed unrepairable, and in three cases after intraoperative failure of ORIF. Three RHA were used: STANDARD in nine cases and RECON in seven cases rHead^®^ SBI-Stryker (Morrisville, PA, USA), and bipolar Judet CRF II^®^ (Tornier SA, Saint-Ismier, France) in three cases. The surgical technique was the same for the different implants: RH was cut a few millimeters under the distal part of the sigmoid notch. Progressive larger rasps were used to open the medullary canal to reach the right size of the prosthesis. Trials were done before definitive implantation.

### Postoperative protocol and follow-up

Postoperatively, patients had a sling for two to four weeks. Active and passive motion were allowed without strength during the first three months. Patients were evaluated at regular intervals (1 month, 3 months, and then yearly).

### Clinical assessment

Computerized data were retrieved to collect demographics, and intraoperative details. All patients were contacted for a clinical and radiological follow-up by a single observer independent from the initial surgery. Patients were asked about their pain levels on a verbal analogical scale (VAS) and their subjective elbow value (SEV).

The clinical examination was bilateral. Active range of motion (ROM) in flexion-extension and forearm prono-supination were measured with a goniometer. Grip strength was measured using a hydraulic dynamometer (JAMAR^®^, Jackson, MI, USA) as well as forearm rotation strength (Inc. Elmsford, NY, USA). The mean measurement of three repeated trials on each side was reported. Elbow stability was assessed by dynamical maneuvers in valgus and varus.

The clinical examination allowed the calculation of two functional scores: the Mayo Elbow Performance Score (MEPS) [10]; and the Lyon Elbow Score (LES) assessing function, ROM and strength in flexion-extension, but also forearm prono-supination [11]. Quick Disabilities of the Arm, Shoulder and Hand (QuickDASH) [12] and Oxford Elbow Score (OES) were calculated [13]. All scores were quantified on a 100-point scale, 100 being the best score for SEV, MEPS, and LES; and 0 for QuickDASH and OES.

Failures were defined as surgical re-intervention if associated to symptomatic discomfort, including secondary resection of the RH with implant or material removal. All complications were recorded. Elbow was considered stiff if active ROM did not reach the functional level defined by Morrey et al. (lack of extension ≥ 30° and/or flexion ≤ 130°) [14].

### Radiographic assessment

**Fig. 2:**
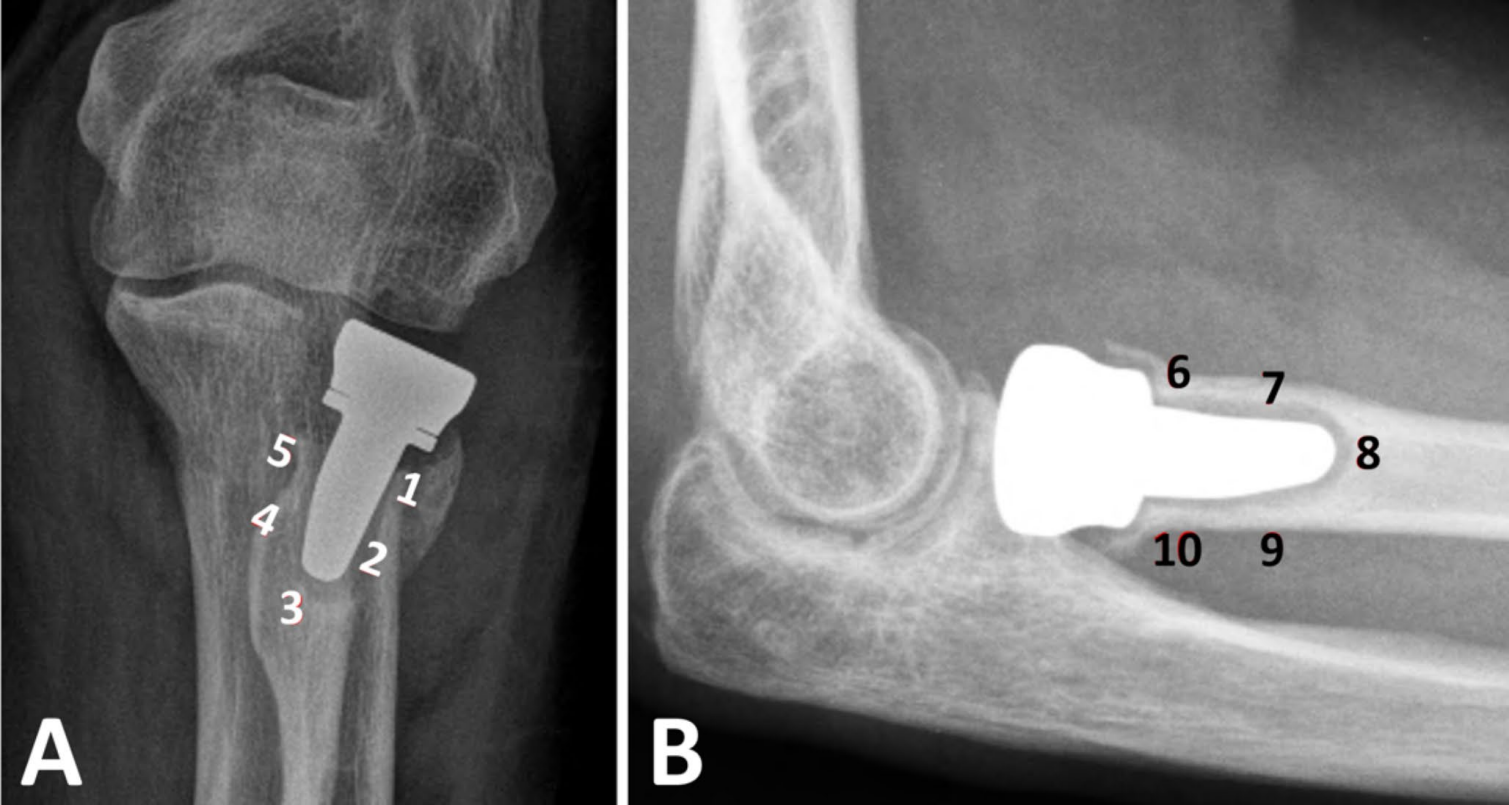
Radiological location of peri-prosthetic radiolucencies. Division into 5 zones in (**A**) anteroposterior view (zones 1 to 5) and (**B**) lateral view (zones 6 to 10) respectively, according to Chen et al. [17]

**Fig. 3:**
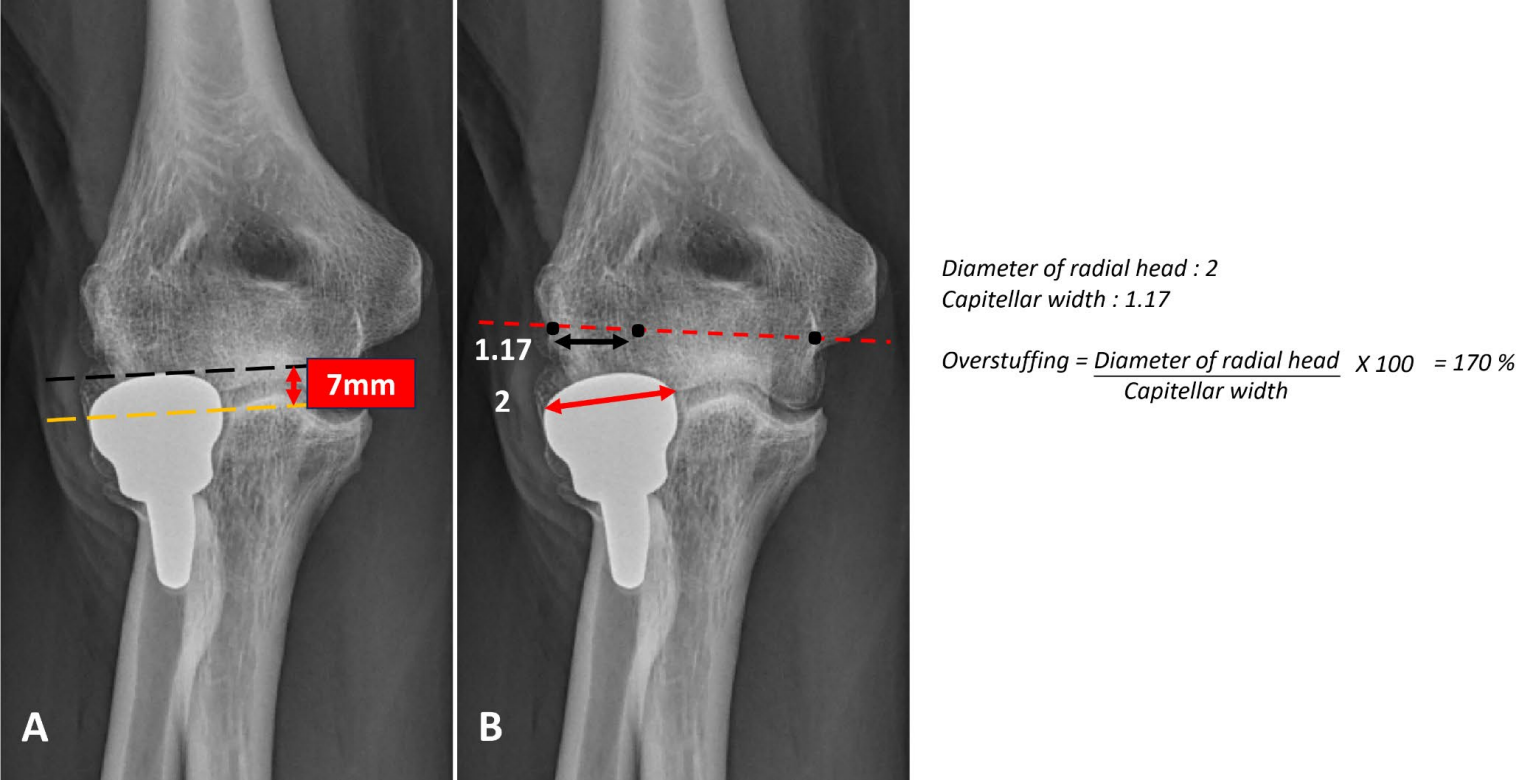
Antero-posterior view of right elbow radiograph 13 years after RHA. Distinguish overlengthening (**A**) and overstuffing (**B**). Note erosion of capitulum with stable elbow (type IIB of Wegmann [18]). Subjective Elbow Value 80 and Lyon Elbow Score 81. (**A**) Overlengthening of 7 mm between the proximal edge of the lesser sigmoid notch (yellow dotted line) and radial head (black dotted line). (**B**) Overstuffing measured by dividing the diameter of the radial head (red arrow) by the capitellar width, the line joining the center of the capitulum and the trochlear groove (red dotted line) [19]

At last follow-up, antero-posterior and lateral views were performed. In both groups heterotopic ossifications (HO) [15] and degenerative changes [16] were recorded. In group RHA, location of peri-prosthetic radiolucencies was reported as described by Chen et al. [17] (Fig. 2). Overlengthening was measured on the postoperative frontal view as described by Wegmann et al. [18], which differs from overstuffing. RHA was considered overstuffed when its diameter exceeded 140% of the native capitulum since RH is anatomically 120–140% larger than the capitulum [19, 20] (Fig. 3).

The number of RH fragments was retrospectively calculated on the preoperative CT-scan.

### Statistical analysis

Statistical analysis was performed using the XL-STAT software (Addinsoft, Paris, France). Continuous variables were reported as mean. Categorical outcomes were compared using Fisher’s exact test and chi-squared test. Normally distributed continuous variables were compared using the Student t-test. A p-value < 0.05 was considered statistically significant for all analyses. A post hoc power analysis was conducted using G*Power software (Dusseldorf, Germany).

### Ethics

This retrospective study involving human participants was in accordance with the ethical standards of the institutional and national research committee and with the 1964 Helsinki Declaration and its later amendments or comparable ethical standards. The Institutional Review Board (IRB) approved this study in our institution on June 2024 (declaration 00012836). An information note and a non-opposition statement have been provided to patients.

This research received no funding.

## Results

### Patient characteristics

In group RHA, a higher proportion of females was observed, and patients were significantly older (51 years vs. 41 years). There were more smokers in group ORIF. The fracture was more often caused by a direct injury in group RHA. For all other characteristics, both groups were comparable (Table 1).

**Table 1:** Outcomes, complications and failures of RHA and ORIF for treatment of isolated Mason Type-III fractures

	RHA (*N* = 19)	ORIF (*N* = 35)	*p* value
Age	51 [26–78]	41 [20–67]	**0.02**
Female	12 (63%)	7 (20%)	**0.002**
Dominant side injured	8 (42%)	15 (42.8%)	0.96
Smoker (Yes)	3 (15.8%)	15 (42.8%)	**0.03**
Mechanism of injury			
Direct trauma	16 (84.2%)	14 (40%)	**< 0.001**
High Energy	9 (47.4%)	14 (40%)	0.6
Manual worker	4 (21%)	14 (40%)	0.15
Work-related injury	5 (26%)	16 (45.7%)	0.16
Follow-up (years)	8.5 [5–13]	7.8 [5–14]	0.4
Flexion (°)	132 [120–140]	134 [120–140]	0.34
Extension (°)	6.5 [0–20]	5.4 [0–30]	0.56
ROM F/E (°)	125.5 [110–140]	128.5 [95–140]	0.36
Pronation (°)	76.7 [60–85]	71.3 [20–85]	**0.04**
Supination (°)	76 [20–90]	69.3 [10–90]	0.17
ROM P/S (°)	152.6 [90–175]	140.6 [30–175]	0.08
VAS	1.2 [0–6]	1.7 [0–7]	0.26
Grip strength recovery (%)	85.8 [40–100]	78.9 [15–100]	0.19
Strength P/S (% to contralateral)	78 [20–115]	78.2 [19–100]	0.96
SEV	81.3 [40–100]	80.4 [35–100]	0.85
LES	79.8 [44–100]	82.8 [44–100]	0.46
MEPS	83.8 [50–100]	82 [44–100]	0.68
QuickDASH	16 [0–41]	12.4 [0–66]	0.33
OES	11 [0–50]	12.5 [0–73]	0.71
Delay to return to work (months)	7.6 [3–12]	6 [1–36]	0.38
Return to work	90%		94%
Physiotherapy duration (months)	9.6 [5–18]	8 [1.5–36]	0.31
Satisfaction (number)	17 (89.5%)	33 (94.3%)	0.56
Satisfaction (/100)	85.5 [40–100]	82.8 [40–100]	0.56
*Complications*			
Stiffness	4 (21%)	4 (11.4%)	0.39
VAS ≥ 4	1 (5.2%)	5 (14.3%)	0.26
CRPS	5 (26.3%)	3 (8.6%)	0.15
*Failures*	2 (10.5%)	3 (8.6%)	0.82

*RHA: radial head arthroplasty*,* ORIF: open reduction and internal fixation*,* Min: Minimum*,* Max: Maximum*,* ROM: range of motion*,* F/E: flexion-extension*,* P/S: prono-supination*,* VAS: Verbal Analogical Scale*,* SEV: Subjective elbow Value*,* LES: Lyon elbow score*,* MEPS: Mayo Elbow Performance Score*,* OES: Oxford Elbow Score. VAS: Verbal Analogical Scale*,* CRPS: Complex regional pain syndrome*

In group RHA, 11 patients (58%) presented RHF with three fragments and eight patients had at least four fragments. In group ORIF, 31 patients (85%) presented RHF with three fragments, and four patients with four fragments (*p* = 0.02). Among these four patients, two had ORIF plating, and the 33 other patients had cannulated screws.

### Clinical and functional outcomes

Clinical and functional outcomes showed no statistical differences (Table 1), except a better pronation in group RHA. The two patients with ORIF plating showed mean flexion-extension 120°, mean pronation 57° and mean supination 25°. One of them achieved good functional outcomes (SEV 70, LES 75, but excellent MEPS 100); the other one presented poor outcomes (SEV 35, LES 44, MEPS 46).

### Complications and failures

No statistical differences were found in both groups. No instability or infection was reported (Table 1).

**Fig. 4:**
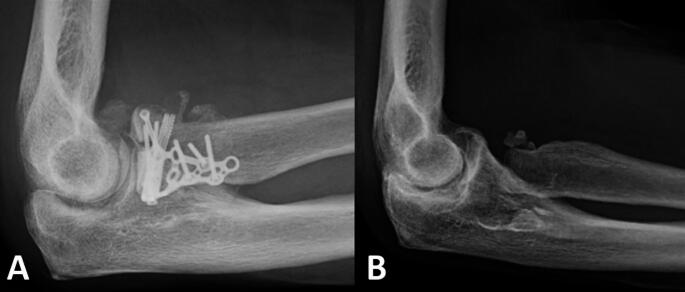
Lateral views of failed ORIF plating. (**A)** at 6 months of the initial surgery; and (**B**) 5 years after the plate removal, radial head resection and arthrolysis. Note heterotopic ossifications on (**A**) and degenerative changes on (**B**). Subjective Elbow Value 35, Flexion/Extension arc: 100°, Prono-supination arc: 120°

Failures occurred in two cases in group RHA and in three cases in group ORIF. In group RHA, implant removal was performed in both cases for aseptic loosening, at four and 65 months. The failed RHA presented satisfactory outcomes at last follow-up (SEV 75, ROM 125° F/E and 150° P/S, 70% strength recovery). In group ORIF, three patients had a reoperation: one for plate and HO removal because of limited forearm rotation at six months. The two others had a secondary RH resection for malunion and presented poor functional outcomes (SEV 40, ROM 97° F/E and 120° P/S, 20% strength recovery) (Fig. 4).

### Radiological outcomes

More HO were reported in group ORIF. More degenerative changes were seen in group RHA, mostly on the capitulum (about 90% in both groups).

**Fig. 5:**
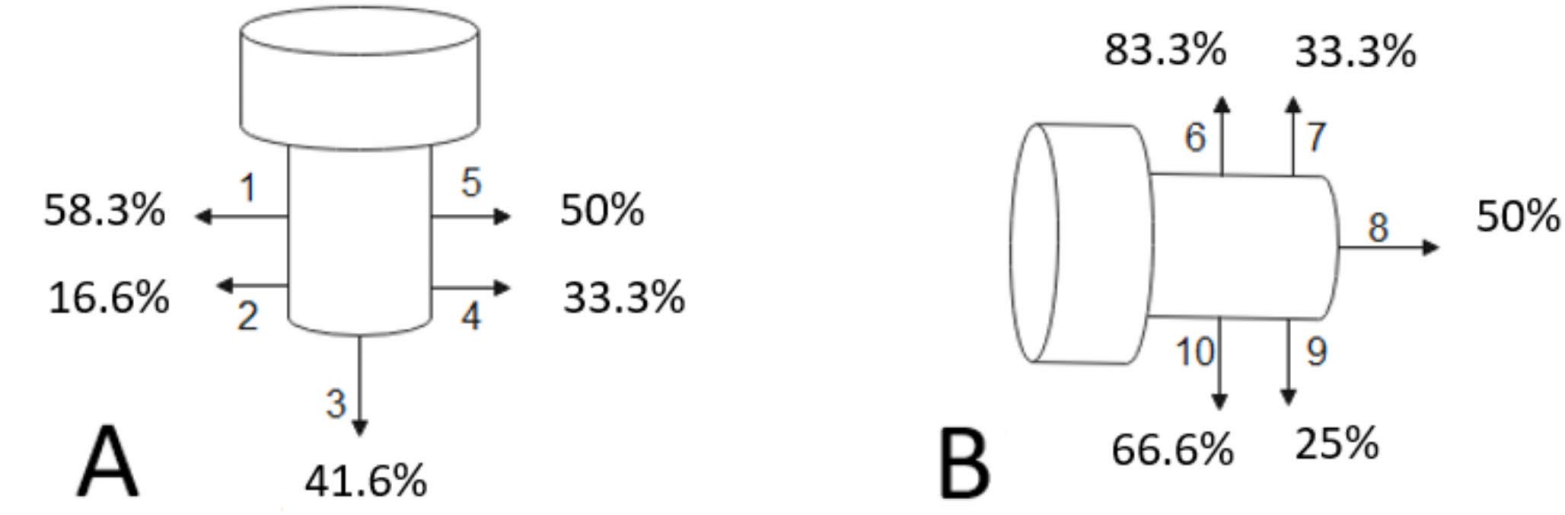
Distribution of periprosthetic radiolucencies in the current study(**A**) antero-posterior and (**B**) lateral views

There was 21% of overstuffing and 42% of overlengthening in group RHA (Table 2). All the overlengthening were type-II (> 2 mm with a stable articulation). Five of them (63%) had erosion of capitellar subchondral bone (Type-IIB) (Fig. 3). Periprosthetic radiolucencies were recorded in 63% and were mainly under the baseplate (Fig. 5).

**Table 2:** Radiological outcomes of RHA and ORIF for treatment of isolated Mason Type-III fractures

	RHA (*N* = 19)	ORIF (*N* = 35)	*p* value
Heterotopic ossification [15], total	3 (15.8%)	15 (42.8%)	**0.03**
Type I: without functional limitation	2 (66.6%)	5 (33.3%)	0.93
Type IIA: limitation in F/E	1 (33.3%)	3 (20%)	0.73
Type IIB: limitation in P/S	0	4 (26.7%)	**0.04**
Type IIC: limitation in both plane	0	3 (20%)	0.08
Type III: ankylosis of forearm, elbow or both	0	0	
Degenerative changes [16], total	13 (68.4%)	10 (28.5%)	**0.005**
Capitulum	12 (92.3%)	9 (90%)	
Humero ulnar	1 (7.6%)	2 (20%)	
Radiolucencies	12 (63%)	0	
Overstuffing	4 (21%)	NA	
Overlengthening [18]	8 (42%)	NA	
Malunion	NA	2 (5.7%)	
Non-union	NA	0	

Results are presented as counts (percentage). RHA: radial head arthroplasty, ORIF: open reduction and internal fixation, F/E: flexion-extension, P/S: prono-supination, NA: Not applicable

### Post hoc power analysis

Given the difference in the number of patients between the two groups, to ensure the reliability of the outcomes, a post hoc power analysis was conducted and found a power of 80% for a moderate effect size with an alpha risk of 0.05.

## Discussion

RHA was performed when the fracture was deemed unrepairable; worse outcomes were theoretically expected in group RHA, but the current study showed comparable clinical and functional results between RHA and ORIF for isolated Mason type-III fractures at a mean of eight years follow-up. Epidemiological differences between the groups can express some unwillingness in using prosthesis for young manual workers. Both treatments lead to functional ROM in flexion-extension and forearm rotation [14, 21] with a statistically better pronation in group RHA, but not clinically relevant. The functional scores (LES, MEPS) were ranked as good in both groups with relatively low disabilities and good satisfaction.

Most fractures requiring RHA were caused by a direct traumatism (84%) which is not commonly described for RHF [1] and might explain the multi-fragmentary aspect of the fracture in absence of associated injuries, regardless of the trauma energy.

The only reason of RHA failure in this study was symptomatic aseptic loosening, which is consistent with the literature [22]. RHA survival rate in this study (89.5% at 8 years) was comparable to the literature (61–97% at 10 years) [23, 24]. The two removed implants were STANDARD rHead^®^. Implant design might play a role in clinical and radiological long-term outcomes and survival rate [25–27].

Overstuffing was estimated at 21% in our study with no estimated rate in the literature, and overlengthening was higher than in the literature (42% vs. 20% [18]). Three out of the five patients with CRPS presented either overstuffing or overlengthening but no statistical correlation could be shown on that small number of patients. We did not find significant correlation between overstuffing or overlengthening and lack of ROM.

ORIF by plating seemed to limit ROM especially in prono-supination despite respect of the safe zone [28]. Functional scores could have been overestimated by MEPS because it does not focus on ROM in prono-supination. Thus, the LES [11] may be more accurate since it encompasses the prono-supination.

Outcomes of failed ORIF were relatively poor in our study, and malunion was the main cause of failure. Therefore, development of a decision tool would be interesting to assess the risk of ORIF failure, to decide preoperatively which treatment is the most appropriate according to clinical and radiological (number and size of the fragments, displacement of the fracture) characteristics of the patient. Also, conversion to RHA must be considered intraoperatively according to the quality, the stability of the fixation and the anatomical articular reduction.

ORIF group showed more HO with 67% of the patients with limited flexion-extension and/or forearm prono-supination, which could explain the better pronation in group RHA. At eight years follow-up, more degenerative changes were observed in group RHA but did not seem to impact clinical and functional outcomes. This result is consistent with the literature reporting wear of the capitulum at a mean time of eight years after RHA [29]. Regarding radiolucencies, Chen et al. reported the absence of correlation with clinical outcomes [17]. Moreover, radiolucencies can be observed early but mostly stayed asymptomatic and were not worsening over time. The authors reported more radiolucencies around the stem tip [17] while they were mostly under the baseplate in this study. This difference might be explained by the stem design (only smooth stems in Chen’s study vs. smooth and porous stems in our study). Our study showed higher rates of radiolucencies and degenerative changes compared to the literature (respectively 63% vs. 46%, and 68% vs. 23%) but less HO (16% vs. 38%) on series of RHA with at least eight years of follow-up [30].

Conservative treatment of RHF is still often preferred even in comminuted fractures [31], while in 2002 Ring and Jupiter [32] already reported unsatisfactory results of ORIF for RHF with more than three articular fragments. No clinical research has specifically studied outcomes of ORIF for isolated Mason type-III fractures yet. Two studies [6, 8] reported the two years outcomes of ORIF in Mason type-III fractures, with comparable flexion-extension ROM but better MEPS than the current study. However, some patients presented associated injuries, which can constitute bias for clinical outcomes (Table 3).

**Table 3:** Outcomes of RHA and/or ORIF for Mason Type-III fractures in the literature compared to the current study

Year	Author	Type of study	Number	Follow-up (years)	ROM F/E	MEPS	DASH	Heterotopic ossification	Humero-ulnar arthritis	Capitullar Erosion	Overstuffing	Periprosthetic radiolucencies	Comments		
RHA															
	Current study	Retrospective	19	8.5	125	83.8	16	15.8%	7.6%	63%	42%	63%	Type prosthesis: Mix		
2019	Laun et al. [7]	Retrospective	10	5.6	119	94	19	35%	35%	27%	0	0	CRF II		
ORIF															
	Current study	Retrospective	35	7.8	128	82	12	42.8%	5.7%	25.7%	NA	NA			
2007	Nalbantoglu et al. [8]	Retrospective	18	23	131	87	-	-	-	-	NA	NA	4 patients: other elbow injuries		
2007	Koslowky et al. [6]	Prospective	12	2	125	97	-	-	-	-	NA	NA	3 patients: other elbow injuries		
Comparative studies															
	Current study	Retrospective	54	8											
2011	Chen et al. [33]	Prospective	45	2.8	-	92 (RHA) vs. 72 (ORIF)		0 (RHA) vs. 8.7% (ORIF)	Complications: 13.6% (RHA) vs. 47.9% (ORIF)				Recommend RHA. Associated injuries		
2009	Ruan et al. [34]	Prospective	22	1.3	Good and excellent Broberg-Morrey scores for RHA (92.9% vs. 12.5% for ORIF)								Recommend RHA		

NA: Not applicable

Laun et al. reported the outcomes of ten RHA for isolated Mason type-III fractures at 5.6 years of follow-up [7]. The authors found comparable flexion-extension ROM and QuickDASH but better MEPS. Their radiological findings slightly differ from the current study.

To our knowledge, only two studies compared RHA and ORIF for Mason type-III fractures [33, 34]. They both were prospective studies with short-term results and recommended RHA in this indication. Both studies did not reach the recommended 3.25 years follow-up for RHA [35]. One study found more complications in group ORIF, but again, some patients had associated injuries [33]. The other study found better Broberg-Morrey scores, 93% ranked as good and excellent in group RHA against only 12% in group ORIF [34]. Nonunion was seen in group ORIF but Kirschner wires were used for fixation, which might partly explain the bad functional results.

This study is the largest series comparing RHA and ORIF with the longest follow-up in current literature but has several weaknesses that must be considered. This was a retrospective study with a different number of patients in each group, and many different low experienced surgeons [36]. There were more patients lost to-follow-up in group ORIF (15% vs. 5%) but this rate is acceptable in a traumatology study with a minimum of five years follow-up. Two of the implants (rHEAD^®^ and rHEAD recon^®^) had been recalled in 2017 because the “efficiency of the device cannot be guaranteed”. New implants may yield better outcomes for RHA in the future. Indeed, monobloc implants appear to be most appropriate for acute injuries with a risk of elbow instability and are associated with a lower revision rate [27]. Therefore, despite the satisfactory outcomes achieved with the modular bipolar implants used in the current study, monobloc implants would now be our preferred option for isolated RHF Mason type-III when ORIF is not satisfying.

## Conclusion

The current study showed comparable clinical and functional outcomes at eight years of follow-up between RHA and ORIF for isolated RHF Mason type-III. However, outcomes of failed ORIF were relatively poor. We recommend RHA if articular reduction or stability of the fixation is not satisfying. In these cases, RHA represents a good option with satisfactory long-term results even in young patients.

## Data Availability

No datasets were generated or analysed during the current study.
